# Comparison of outcomes of ORIF versus bidirectional tractor and arthroscopically assisted CRIF in the treatment of lateral tibial plateau fractures: a retrospective cohort study

**DOI:** 10.1186/s13018-021-02447-w

**Published:** 2021-05-03

**Authors:** Xiangtian Deng, Hongzhi Hu, Yiran Zhang, Weijian Liu, Qingcheng Song, Xiaodong Cheng, Jian Zhu, Sifan Yang, Zhipeng Ye, Haitao Guan, Boyu Zhang, Zhanle Zheng, Yingze Zhang

**Affiliations:** 1 School of Medicine, Nankai University, Tianjin, 300071 People’s Republic of China; 2 Department of Orthopaedic Surgery of Hebei Province, Third Hospital of Hebei Medical University, 139 Ziqiang Road, Shijiazhuang, 050051 Hebei People’s Republic of China; 3 NHC Key Laboratory of Intelligent Orthopaedic Equipment, Third Hospital of Hebei Medical University, Shijiazhuang, 050051 Hebei People’s Republic of China; 4 Department of Orthopedics, Union Hospital of Tongji Medical College of Huazhong University of Science and Technology, Wuhan, 430022 People’s Republic of China; 5 Orthopaedic Institution of Hebei Province, Shijiazhuang, 050051 Hebei People’s Republic of China

**Keywords:** Bidirectional traction, Lateral tibial plateau fractures, Open reduction and internal fixation, Closed reduction and internal fixation, Arthroscopy

## Abstract

**Background:**

Lateral tibial plateau fractures (TPFs) are often treated with conventional open reduction and internal fixation (ORIF) through standard anterolateral sub-meniscal arthrotomy. There has been increasing support for “bidirectional rapid redactor” device-assisted closed reduction and internal fixation (CRIF) for treating TPFs. The aim of the present study is to compare the clinical and radiological outcomes between CRIF and ORIF procedures.

**Methods:**

We performed a retrospective cohort study of 55 lateral TPF patients (Schatzker types I–III) who accepted surgical treatment at our trauma level 1 center between January 2016 and January 2018. They were divided into the CRIF group (32 patients) and the ORIF group (23 patients) based upon the different surgical protocols. The patients’ clinical outcome analysis was evaluated by using the Knee Society Score (KSS) and Rasmussen’s clinical score. For radiological assessment, changes in tibial plateau width (TPW), articular depression depth (ADD), medial proximal tibial angle (MPTA), and posterior tibial slope angle (PTSA) were evaluated using radiographs and computed tomography (CT) scan.

**Results:**

The CRIF group had a mean follow-up of 28.9 months, and the ORIF group had a mean follow-up of 30.7 months (*p*>0.05). Furthermore, there was no statistically significant difference in terms of age, gender, injury mechanism, follow-up time, time interval from injury to surgery, and Schatzker classification in the two groups. With respect to the clinical outcomes including the KSS score and Rasmussen’s clinical score, there was also no significant difference (*p*>0.05). Nevertheless, the CRIF group had lower intra-operative blood loss, shorter hospitalization days, and better range of movement of the knee joint than the ORIF group (*p*<0.05). Furthermore, CRIF had better radiological results when compared to the ORIF group using Rasmussen’s radiological score (*p*<0.05), although no significant difference was observed in TPW, ADD, MPTA, and PTSA between the two groups (*p*>0.05).

**Conclusion:**

The present study showed that CRIF could achieve comparable clinical outcomes and better radiological results for treating lateral TPFs as compared with conventional ORIF.

## Background

Tibial plateau fractures (TPFs), accounting for approximately 1.6% of all adult fractures in China [1], are complex intra-articular fractures that typically involve either active young adults caused by a high-energy trauma or elderly patients with osteoporosis who sustain low-energy injuries. The key aspect of treatment for these fractures is not only requiring restoration of the lower limb mechanical axis and anatomic reduction of the articular surface, but also minimizing complications and achieving functional ability [2, 3]. Much of the more recent literatures regarding different surgical procedures applied to TPFs is conflicting, including open reduction and internal fixation (ORIF), arthroscopically assisted reduction and internal fixation (ARIF), and closed reduction and internal fixation (CRIF) [4–7]. As such, the optimal treatment for lateral TPFs still remains debatable for orthopedic trauma surgeons, especially for patients associated with intra-articular soft tissue lesions such as meniscal tears and/or cruciate ligamentous injuries.

Generally, the traditional ORIF technique requires excessive soft tissue dissection and may increase the risk of complication rates such as wound infection, neurovascular injury, thrombosis, and soft tissue injuries [8–11]. In recent years, many authors have suggested that ARIF can provide a direct exposure visualization of the intra-articular structure, which can diagnose and address concomitant intra-articular soft tissue injuries simultaneously [12–15]. Among many surgical protocols, ARIF was considered as a minimally invasive technique for TPFs for several decades. However, a certain longer learning curve, technical difficulty for fracture reduction, and time-consuming may impede its widespread use for orthopedic trauma surgeons. Furthermore, at many hospitals, arthroscopy is not the preferred and routine approach in the treatment of TPFs in the acute setting.

“Bidirectional traction device”-assisted CRIF for TPFs has been successfully applied in the treatment of bicondylar TPFs (Shcatzker V–IV) with advantages of less trauma, lower complication rates, and anatomical restoration of articular congruity [6]. Despite these advantages of CRIF assisted by bidirectional traction, some difficulties also existed in treating concomitant intra-articular meniscal tears and cruciate ligamentous injuries, which may lead to earlier onset of post-traumatic knee osteoarthritis if these accompanying soft tissue injuries were overlooked in clinical practice. Hence, an arthroscopic examination was performed immediately after CRIF in the present study, which might be helpful in the management of concomitant soft tissue injuries.

To our knowledge, however, the present study is the first to compare the clinical and radiological outcomes between CRIF and ORIF to date. It was our hypothesis that patients with lateral TPFs treated by CRIF had comparable clinical outcomes and better radiological results than ORIF.

## Methods

### Patients

This study was designed as a retrospective cohort study, and the work complied with the Strengthening the Reporting of Cohort Studies in Surgery (STROCSS) criteria [16]. This study was approved by the Ethnical Committee of the Third Hospital of Hebei Medical University and in accordance with the Declaration of Helsinki. A total of 55 lateral TPF patients (Schatzker I–III) treated either by CRIF or ORIF at the trauma level 1 center of our hospital were retrospectively reviewed from January 2016 to January 2018. Our inclusion criteria for patients were as follows: aged older than 18 years at the time of surgery, closed lateral TPFs, time interval from injury to surgery less than 21 days, and follow-up time more than 24 months. The exclusion criteria were as follows: open and/or pathological fractures, associated with peri-articular fracture of the knee joint, Schatzker IV–VI, multiple fractures and/or polytrauma, and incomplete patient data.

### Surgical procedure

**Fig. 1:**
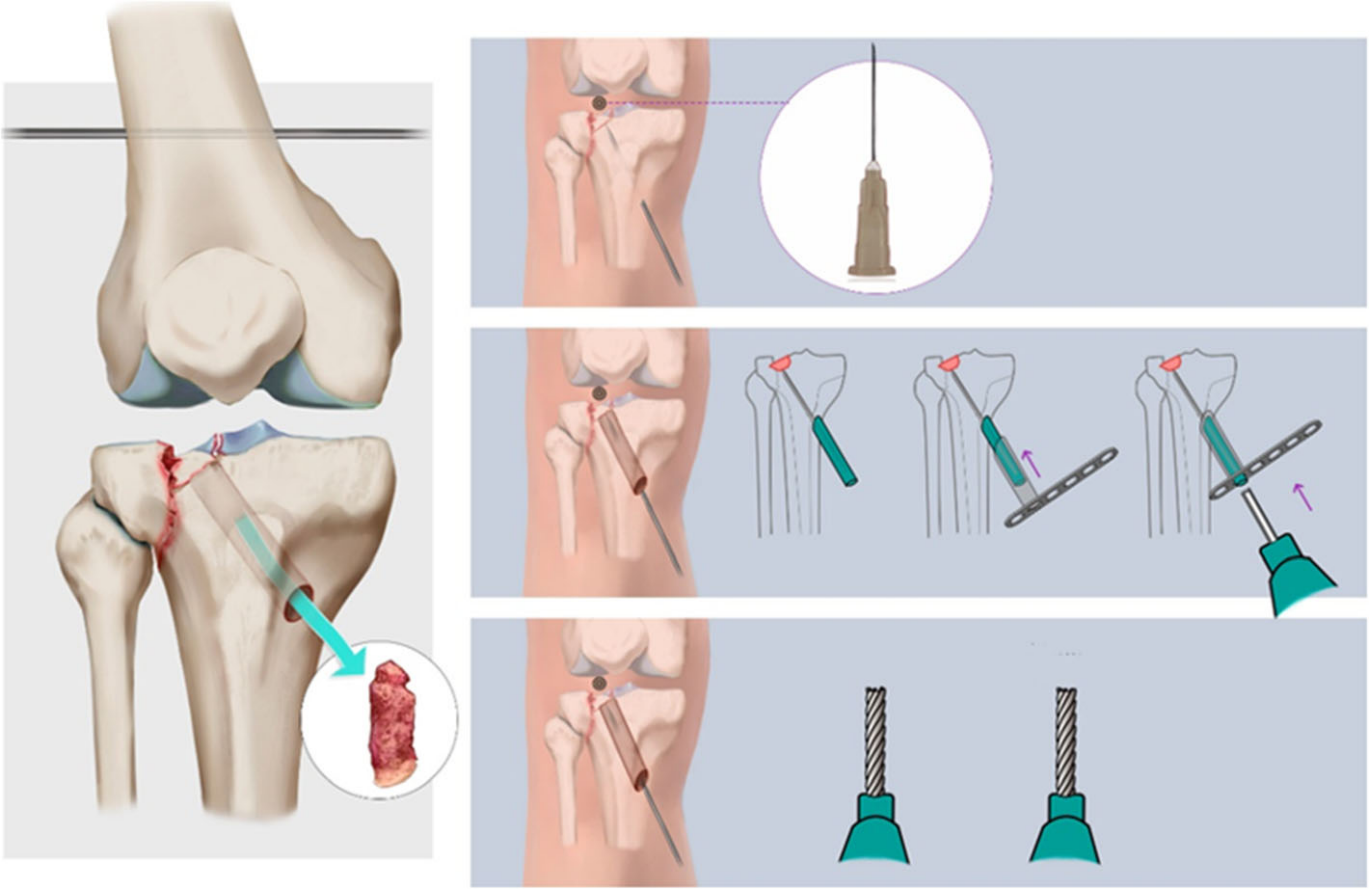
Illustration of the surgical technique in closed reduction for patients with articular surface depression using iliac crest bone graft from the metaphyseal window

Patients were placed in a supine position with the knees maintained in a flexed 30° position under general anesthesia. A pneumatic tourniquet was used at the proximal thigh for all patients. The surgical technique assisted by “bidirectional traction” device for treating TPFs has been recently reported in our literatures [16]. In the CRIF group, the “bidirectional rapid reductor” following a mechanical closed-loop system was applied to reduce and maintain the displaced spilt fracture fragment through enormous traction force and surrounding soft tissue compression. For patients with a depressed articular surface, a small metaphyseal window was performed using a bone impactor to reduce and elevate the depressed fracture to the original articular surface under the assistance of a C-arm fluoroscope. Meanwhile, the remaining metaphyseal bony defects were then filled with iliac crest bone graft, stabilizing and maintaining the restored articular level (Fig. 1). Instead, in the ORIF group, a standard anterolateral sub-meniscal arthrotomy was used to restore the depressed articular surface under direct visualization and to confirm that it was achieved to the satisfaction of the surgeon. Furthermore, the reduction technique is the same as the CRIF group. After that, minimally invasive percutaneous plate osteosynthesis (MIPPO) was performed using a locking compression plate (LCP) applied to the lateral cortex.

Importantly, in the CRIF group, arthroscopic examination was performed immediately to evaluate the quality of fracture reduction and diagnose and address the concomitant intra-articular structure injuries. For instance, in cases of lateral and/or medial meniscal injuries associated with TPFs, a suture repair or partial meniscectomy was performed simultaneously. In patients with cruciate ligamentous tears, second-stage ligamentous reconstruction would be considered if post-operative knee instability still exists.

### Clinical outcomes and radiographic evaluation

Patients were evaluated in terms of age, gender, time interval from injury to surgery, mechanism of injury, type of Schatzker fracture, range of movement (ROM), clinical outcomes, and radiological parameters. All patients underwent X-rays and computed tomography (CT) scan of their injured knees pre-operatively. CT scan was performed to evaluate the fracture type according to the Schatzker classification [17]. All the operations were performed by the same team at our institution. The operative indications for TPFs included articular step-off exceeding 3 mm, condylar widening greater than 5 mm, or malalignment greater than 5° [18].

**Fig. 2:**
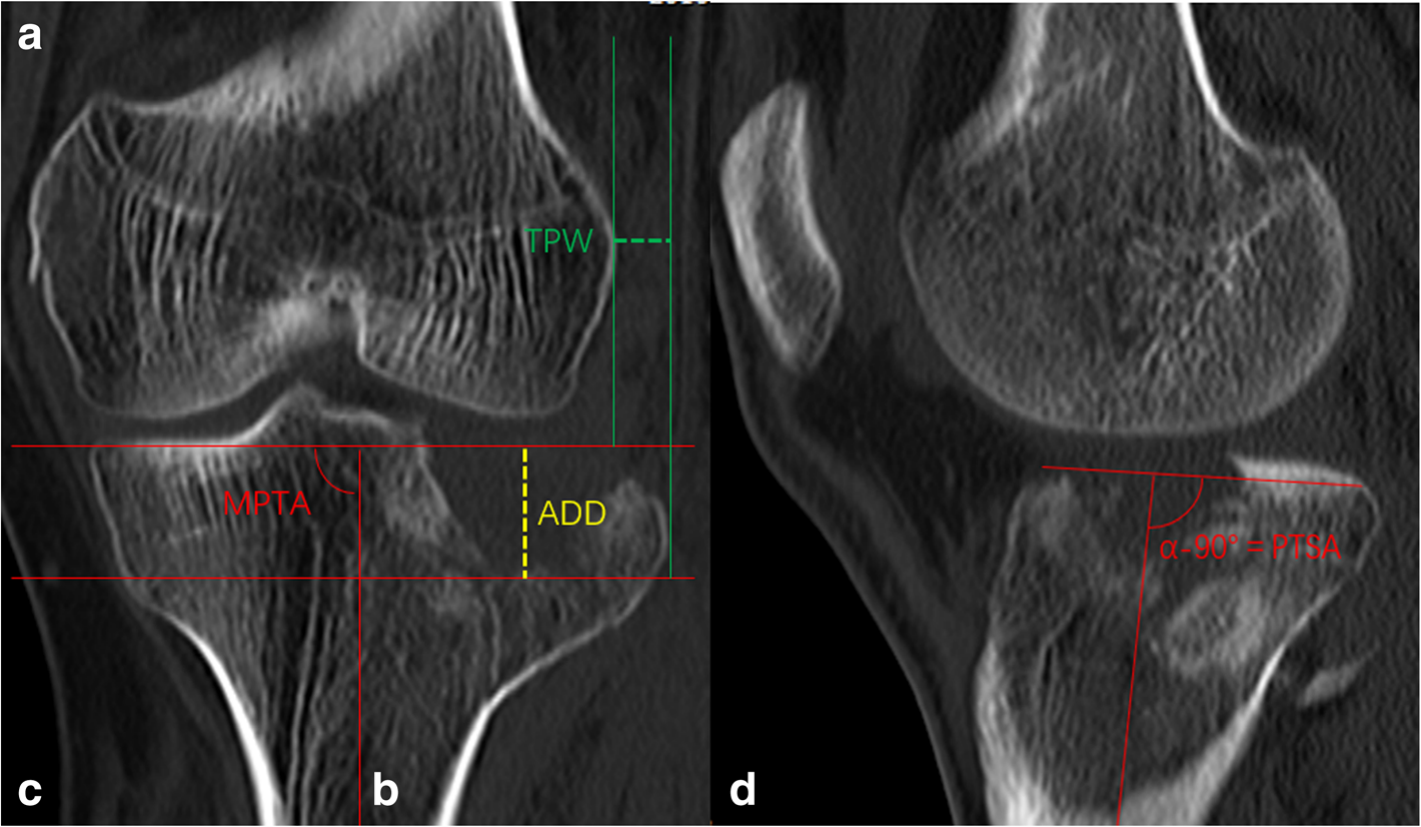
Radiological measurement of the TPW, ADD, MPTA, and PTSA. **a** The TPW (green dash line) was between the tangential line to the lateral femoral condyle and the tangential line to the widest edge of the lateral tibial plateau. **b** The ADD (yellow dash line) was measured between the tangential line to the articular surface and the tangential line to the lowest point of depression. **c** The MPTA was defined as the medial angle between the tibial anatomic axis and the joint line of the proximal tibia. **d** The PTSA is measured as the angle between the line drawn along the anterior and posterior edges of the tibial plateau and the anatomical axis of the tibial. TPW, tibial plateau width; ADD, articular depression depth; MPTA, medial proximal tibial angle; PTSA, posterior tibial slope angle

**Fig. 3:**
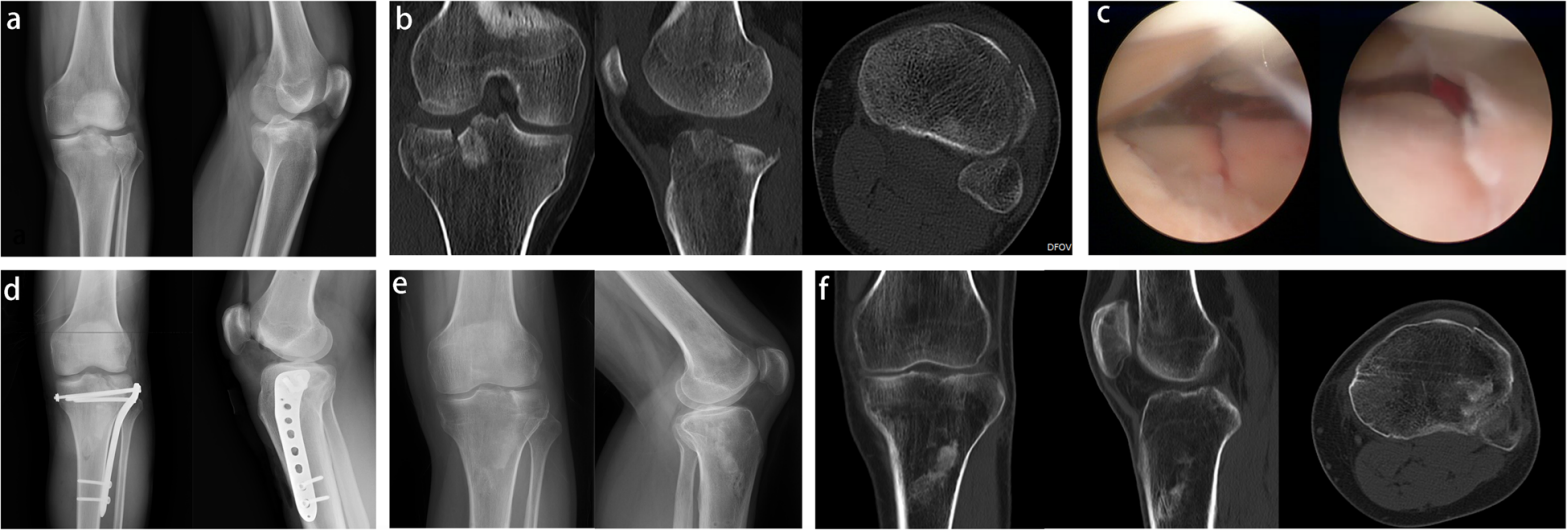
A 43-year-old male patient suffering from a Schatzker II fracture, treated with CRIF and arthroscopic examination. **a** Pre-operative radiograph. **b** Pre-operative CT scan. **c** Arthroscopic examination of the articular surface after reduction. **d** Post-operative radiographs immediately. **e**, **f** Post-operative radiographs and CT scan at the final follow-up

The Knee Society Score (KSS) and Rasmussen’s clinical score were used to evaluate the patients’ clinical outcomes. Radiological parameters, including tibial plateau width (TPW), articular surface depression depth (ADD), medial proximal tibial angle (MPTA), and posterior tibial slope angle (PTSA), were measured and analyzed from the radiographs and CT scan. The radiological measurements of TPW, ADD, MPTA, and PTSA are shown in Figs. 2 and 3.

### Statistical analysis

The SPSS software (version 24.0, IBM Corp., USA) was used for the statistical analysis. Continuous variables were recorded as mean ± standard deviation. To determine the number of test patients, the sample size was calculated under a significance level of 0.05 and a power of 0.80. As a result, each group required 20 patients. Categorical variables that presented as percentages were determined by the chi-square test or Fisher exact test. The Student *t* test was performed for statistical analysis of clinical outcomes and radiological parameters between the two groups, and *p* value < 0.05 was considered statistically significant for all tests.

## Results

### Patient demographics

A total of 55 lateral TPF patients treated surgically were enrolled in the present study, and they were divided into the CRIF group and ORIF group, as summarized in Table 1. No secondary reduction loss was observed in all patients. There were 42 (76.4%) males and 13 females (23.6%), with a mean age of 45.9 years at the time of surgery. Of the included patients, the CRIF group comprised 32 (58.2%) patients with a mean age of 44.6 years, while the ORIF group comprised 23 (41.8%) patients with a mean age of 47.8 years. There was no statistical difference in terms of age (*p*=0.379), gender (*p*=0.099), injury mechanism (*p*=0.315), follow-up time (*p*=0.187), time interval from injury to surgery (*p*=0.129), and type of Schatzker fracture (*p*=0.593) in the two groups (*p*>0.05). In addition, there were 20 patients associated with intra-articular lesions, of whom 17 in the CRIF group and 3 in the ORIF group, as presented in Table 1.

**Table 1:** The demographic date of the two groups

Characteristics	CRIF group	ORIF group	*p* value
Number of patients	32	23	–
Age (years)	44.6±11.9	47.8±14.9	0.379
Gender (*n* (%))			0.099
Male	27 (84.4%)	15 (65.2%)	
Female	5 (15.6%)	8 (34.8%)	
Injury mechanism (*n* (%))			0.315
Vehicle accident	21 (65.6%)	12 (52.2%)	
Falling	11 (34.4%)	11 (47.8%)	
Associated lesions			N/A
Meniscal tears	14	3	
ACL tears	3	N/A	
Follow-up time (months)	28.9±3.1	30.7±6.7	0.187
Time interval from injury to surgery (days)	6.7±2.2	7.7±2.6	0.129
Schatzker classification (*n* (%))			0.593
Schatzker I	4 (12.5%)	2 (8.7%)	
Schatzker II	19 (59.4%)	17 (73.9%)	
Schatzker III	9 (28.1%)	4 (17.4%)	

The values are presented as the mean±SD or *n* (%)

*CRIF* closed reduction and internal fixation, *ORIF* open reduction and internal fixation, *ACL* anterior cruciate ligament, *N/A* not available

### Comparison of clinical outcomes

The patients’ clinical outcomes are demonstrated in Table 2. We identified that there was a statistically significant difference concerning the intra-operative blood loss (*p*=0.027), duration of hospitalization (*p*=0.003), and range of motion (*p*=0.014) between the CRIF group and ORIF group (*p*<0.05). Furthermore, the mean KSS score and Rasmussen clinical score between the two groups were comparable at the final follow-up, and no statistically significant difference was observed between the two groups of patients (*p*>0.05).

**Table 2:** Clinical outcomes of the two groups

Characteristics	CRIF group	ORIF group	*p* value
Operating time (min)	134.8±36.9	123.5±37.1	0.269
Intra-operative blood loss (ml)	145.6±52.8	181.7±64.8	0.027*
Duration of hospitalization (days)	4.9±1.2	7.1±3.8	0.003*
ROM (°)	117.3±10.5	109.6±11.8	0.014*
Complications			0.003*
Infection	0	1	
DVT	0	3	
Stiffness	0	1	
Neural palsy	0	1	
Instability	0	0	
Compartment syndrome	0	0	
KSS score			
Post-operative	81.5±3.7	80.7±2.9	0.392
Last follow-up	84.6±5.4	83.1±6.2	0.344
Rasmussen’s clinical score			
Post-operative	25.2±2.2	24.8±2.4	0.525
Last follow-up	27.1±2.8	26.2±2.4	0.198

The values are presented as the mean±SD or *n* (%)

*ROM* range of movement, *KSS* Knee Society Score, *DVT* deep venous thrombosis

*Statistically significant difference between the two groups (*p*<0.05)

The overall complication rate was 10.9% (6/55). It was noted that no complication occurred in the CRIF group while all post-operative complications occurred in the ORIF group, showing a significant difference between the two groups with regard to complication rates (*p*<0.05). One case of infection improved successfully by antibiotic treatment. Three patients with deep venous thrombosis were managed by physical therapy. Furthermore, in one case, stiffness and neural palsy were successfully treated with manipulation under general anesthesia and physical therapy, respectively.

### Comparison of radiological measurements

The radiological results are summarized in Table 3. At the last follow-up, no evidence of secondary displacement and post-traumatic osteoarthritis was identified in both groups. All patients had healed their fractures. The mean Rasmussen’s radiological scores were 14.2±2.4 and 12.7±1.8 in the CRIF group and ORIF group, respectively, which showed a significant difference (*p*<0.05). Furthermore, the mean pre-operative TPW was 4.4±3.7 mm in the CRIF group, while that of the ORIF group was 4.3±2.8 mm, being decreased to 1.7±1.5 mm and 1.8±1.6 mm, respectively. Post-operative correction was obvious in both groups, with a statistically significant difference in the width of the tibial plateau (*p*<0.05). At the final follow-up, the TPW was 2.2±1.2 mm and 2.4±2.1 mm, respectively, showing similar post-operative TPW in the two groups (*p*>0.05). The mean pre-operative ADD was 12.4±5.7 mm in the CRIF group and 11.8±5.4 mm in the ORIF group, which was significantly decreased to 2.1±1.3 mm and 1.7±1.3 mm after surgery, respectively. At the final follow-up, the ADD was 2.5±1.9 mm and 2.1±1.8 mm, respectively. The mean MPTA was 92.7°±2.3° pre-operatively, 89.2°±1.8° post-operatively, and 88.6°±1.5° at the final follow-up in the CRIF group, while those of the ORIF group were 92.3°±1.9° pre-operatively, 88.7°±2.2° post-operatively, and 87.9°±1.7° at the final follow-up. The mean PTSA was 12.8°±3.7° pre-operatively, 9.6°±3.1° post-operatively, and 10.6°±2.9° at the final follow-up in the CRIF group, while those of the ORIF group were 13.2°±2.8° pre-operatively, 9.8°±4.3° post-operatively, and 10.4°±2.5° at the final follow-up.

**Table 3:** Comparison of radiological parameters between the groups

Variables	CRIF group	ORIF group	*p* value
Rasmussen’s radiological score	14.2±2.4	12.7±1.8	0.015*
TPW (mm)			
Pre-operative	4.4±3.7	4.3±2.8	0.913
Post-operative	1.7±1.5	1.8±1.6	0.813
Last follow-up	2.2±1.2	2.4±2.1	0.656
ADD (mm)			
Pre-operative	12.4±5.7	11.8±5.4	0.696
Post-operative	2.1±1.3	1.7±1.3	0.423
Last follow-up	2.5±1.9	2.1±1.8	0.745
MPTA (°)			
Pre-operative	92.7±2.3	92.3±1.9	0.498
Post-operative	89.2±1.8	88.7±2.2	0.375
Last follow-up	88.6±1.5	87.9±1.7	0.112
PTSA (°)			
Pre-operative	12.8±3.7	13.2±2.8	0.665
Post-operative	9.6±3.1	9.8±4.3	0.842
Last follow-up	10.6±2.9	10.4±2.5	0.791

The values are presented as the mean±SD or *n* (%)

*TPW* tibial plateau width, *ADD* articular depression depth, *MPTA* medial proximal tibial angle, PTSA proximal tibial slope angle

*Statistically significant difference between the two groups (*p*<0.05)

## Discussion

The most important finding of the present study was that CRIF had comparable clinical outcomes and better radiological results than ORIF. In our study, we identified that the CRIF group had lower intra-operative blood loss, shorter hospitalization days, fewer complication rates, and better ROM of the knee joint, indicating minimally invasive fracture reduction with faster recovery and better functionality when compared to the conventional ORIF technique. However, clinical outcomes were similar concerning the KSS score and Rasmussen’s clinical score, which are in contrast to the findings of a previous study reported by Buckley et al. [19]. They showed that ORIF provided better quality reduction and satisfactory medium-term results for TPFs via a sub-meniscal arthrotomy approach when compared to CRIF. The significant discrepancy of clinical outcomes in patients with tibial plateau fracture may be explained by the fact that additional application of arthroscopy immediately after internal fixation to address concomitant meniscal tears in the CRIF group in the present study.

Actually, arthroscopic management in lateral tibial plateau fractures (Schatzker I–III) is widely used and acceptable. Wang et al. [20] demonstrated that both ARIF and ORIF had satisfactory clinical results for treating TPFs while ARIF had better radiological results than ORIF. Le Baron et al. [21] reported that ARIF and ORIF are both acceptable treatments of TPFs, with no statistically significant differences found in the clinical outcomes and radiological results. In addition, Jeong et al. [22] showed no significant differences in the clinical outcomes between the arthroscopy combined with ORIF procedures and ORIF alone. As is well known, however, arthroscopically assisted anatomical reduction of the articular surface and restoration of the mechanical axis of the lower extremity in TPFs are technically demanding for orthopedic trauma surgeons. Therefore, unlike prior literatures with arthroscopic-assisted fracture reduction, immediate arthroscopy was performed just after CRIF through the standard anteromedial and anterolateral portals in our study, which minimizes the technical difficulty.

Although the application of arthroscopic examination during surgery increased operation time, our study identified that the CRIF group had no complication related to arthroscopy and had better radiological results with Rasmussen’s radiological score than ORIF alone. This is due in part to the fact that a potential advantage of the arthroscopy may provide a better evaluation for quality reduction and treatment for concomitant intra-articular lesions through a less invasive procedure than ORIF. Furthermore, the bidirectional tractor, a reliable and minimally invasive procedure, provided advantages of nearly anatomical reduction of fracture, restoration of articular congruity, and less dissection of soft tissue. Thus, this reinforces the superiority of arthroscopy management combined with CRIF procedure in treating patients with TPFs, which are consistent with the viewpoint of the Canadian Orthopaedic Trauma Society [23].

The targets of the surgical treatment in TPFs are not only the anatomical reduction of articular congruity and restoration of lower limb alignment, but also the reasonable treatment of concomitant intra-articular injuries to allow early rehabilitation, enhancement of knee stability, and minimize the risk of post-traumatic osteoarthritis. In addition, TPFs are often associated with meniscal tears and cruciate ligamentous injuries. Abdel-Hamid et al. [24] arthroscopically evaluated 98 TPFs and identified an incidence of 71% for intra-articular soft tissue injuries. A recent study by Deng et al. [16] identified that the prevalence of concurrent cruciate ligamentous injuries in TPFs was 37.3% following arthroscopy after CRIF. Despite the known relatively high incidence of associated soft tissue injuries in TPFs, the influence of untreated intra-articular soft tissue injuries on clinical outcomes remains unclear. Elsoe et al. [25] identified that the presence of soft tissue injuries associated with TPFs did not significantly affect the clinical outcome. Similarly, Warner et al. [26] also demonstrated that there was no significant difference in the clinical outcomes between the patients with sutured meniscal tears and untreated meniscal tears in TPFs. In contrast to these prior studies, our results have shown that patients with meniscus sutured have a better range of motion than those who did not. Therefore, we concluded that the appropriate management of concomitant meniscal tears in TPFs at the time of surgery may maximize functional restoration and improved patients’ satisfaction.

Several limitations of this present study should also be noted. First, selection bias was inevitable due to the nature of the retrospective study. Second, this study in calculating intra-operative blood loss is not accurate due to the use of arthroscopy with irrigation fluid at the time of fracture fixation. Third, the number of patients was relatively small, and the follow-up time was short, which may not observe the development of post-traumatic osteoarthritis. Fourth, a second-look arthroscopic examination to demonstrate the therapeutic effect of the intra-articular soft tissue injuries in TPFs was absent.

## Conclusion

Our study showed that CRIF had comparable clinical results with ORIF, and it had advantages over ORIF with regard to better radiological results, less trauma, and fewer complication rates. Furthermore, we recommend that arthroscopic examination should be performed after internal fixation to diagnose and address concomitant intra-articular soft tissue injuries.

## Data Availability

All the data and materials involving this article will be available upon request by sending an e-mail to the first author.

## Abbreviations

TPFs: Tibial plateau fractures
ORIF: Open reduction and internal fixation
CRIF: Closed reduction and internal fixation
KSS: Knee Society Score
TPW: Tibial plateau width
ADD: Articular depression depth
MPTA: Medial proximal tibial angle
PTSA: Posterior tibial slope angle
CT: Computed tomography
ARIF: Arthroscopically assisted reduction and internal fixation
MIPPO: Minimally invasive percutaneous plate osteosynthesis
LCP: Locking compression plate
ROM: Range of motion
